# Effects of Loop Nucleobase Substitution on G-Quadruplex Thermal Stability in Aqueous Glycine Betaine, Proline, TMAO, and Urea Solutions

**DOI:** 10.3390/biom16050697

**Published:** 2026-05-08

**Authors:** Jeffrey J. Schwinefus, Marija Corluka, Isabella Dobrinski, Stella L. Jaeckle, Joshua Kim, Grace Knowlan, Hannah Omodt, Noah Otto, Mari V. Reid, Reid Rognerud, Kathryn M. Stein

**Affiliations:** Department of Chemistry, St. Olaf College, Northfield, MN 55057, USA; corluka@vt.edu (M.C.); isabella_dobrinski@hsdm.harvard.edu (I.D.); sjaeckle@mcw.edu (S.L.J.); jmkimfive@gmail.com (J.K.); knowlangrace@gmail.com (G.K.); omodt031@umn.edu (H.O.); otto4@stolaf.edu (N.O.); reidx217@umn.edu (M.V.R.); rogner2@stolaf.edu (R.R.); stein28@uwm.edu (K.M.S.)

**Keywords:** G-quadruplex, glycine betaine, proline, thermal stability, urea, TMAO

## Abstract

G-quadruplexes are guanine-rich DNA or RNA structures comprising two or more stacked guanine tetrads (G-quartet) with nucleobases in the loops linking the G-quartets. The thermal stability of the thrombin-binding aptamer G2 d(G_2_T_2_G_2_TGTG_2_T_2_G_2_) G-quadruplex was quantified using *m*-values in aqueous glycine betaine (GB), proline, TMAO, and urea solutions using UV-absorbance spectroscopy. The thermal stability of the G2 variants was also explored with nucleobase substitutions in the TGT loop (TAT, TCT, TTT, UGU, and UUU), in the T_2_ loops (T_4_, U_2_), or in both loops (UGU and U_2_, UUU and U_2_). GB and TMAO strongly stabilized G2 and its variants. Urea weakly destabilized G2 and its variants, while proline acted as a weak stabilizer or destabilizer depending on G2 variant nucleobase sequence. Both the unfolding enthalpy and entropy were positively correlated with cosolute molality for all cosolute and G-quadruplex combinations. Analysis of the change in solvent-accessible surface area (ΔASA) after unfolding G2 suggested the stability of G2 and its variants in aqueous cosolute solutions was driven by favorable interaction of cosolutes with G-quartet ΔASA and net-favorable or unfavorable interactions with the loop ΔASA. Our results reinforce a general mechanism of folded DNA and RNA stability modulation in cosolute solutions.

## 1. Introduction

G-quadruplexes are non-canonical DNA or RNA structures comprising one or more nucleic acid single strands with at least two stacked guanine tetrads (G-tetrads) [1,2,3,4,5,6,7,8]. The O6 oxygens on the G-tetrad guanine nucleobases coordinate with monovalent cations such as potassium or sodium ions, helping to stabilize the folded structure [1,3,6]. Each guanine nucleobase in a G-tetrad interacts with two adjacent guanines through Hoogsteen hydrogen bonding, forming a coplanar structure while a single stabilizing cation is sandwiched between two G-tetrads [1,3,6]. The sequences of nucleobases that are not incorporated in the G-tetrads are referred to as loops. G-quadruplex folding stability is sensitive to loop length and sequence due to hydrogen bonding and nucleobase stacking within the loops and between the loops and the G-tetrads [9,10,11,12,13,14].

G-quadruplex-forming sequences are ubiquitous in the human genome, where they are found to play crucial roles [1,5,15]. G-quadruplexes participate in multiple cellular functions including chromatin and transcription regulation, DNA recombination, and protein translation [3,5,15]. Formation of G-quadruplexes in chromosomal telomeric regions inhibits telomere elongation [1,16], making telomeric G-quadruplex sequences potential targets for anticancer therapeutics [17,18,19]. In addition, the formation of G-quadruplexes in chromosomal DNA has been linked to neurodegenerative diseases [5,15,20]. Finally, some DNA aptamers that bind to specific cellular targets are known to form G-quadruplexes [9,21,22]. Numerous computational [23,24,25] and in vitro experimental studies [12,13,26,27,28,29,30,31,32] have focused on the dependence of G-quadruplex structural stability on environmental conditions.

Cosolutes (also referred to as osmolytes) are naturally occurring, low-molar-mass organic compounds including urea, glycine betaine (GB), the amino acid L-proline, and trimethylamine *N*-oxide (TMAO) [33]. The cellular accumulation of cosolutes is an important adaptation of organisms living in high-temperature, high-salinity, or high-pressure environments [33,34,35] that can have dramatic influences on the stability of macromolecules such as proteins, nucleic acids, and cellular membranes as well as ligand binding and enzyme kinetics [33,34,35,36,37,38,39,40,41,42,43]. Cosolutes may also play a role in the liquid–liquid phase separation condensation of proteins, leading to neurodegenerative disorders and attenuating cancer progression pathways [35,44,45,46].

Urea is an established protein and nucleic acid denaturant. Urea preferentially accumulates at the protein amide backbone and hydrocarbon solvent-accessible surface areas (SASAs) and the DNA or RNA nucleobase SASAs which are exposed during unfolding [40,47]. G-quadruplex thermal stability decreases with urea concentration, although the degree of destabilization is dependent on the DNA sequence [12,13,23,27,29,30,31,32]. Urea can stabilize G-quadruplexes with single-base loops [13]. Alternatively, TMAO stabilizes protein structure and has the ability to offset the denaturing effects of urea [28,37]. TMAO also stabilizes or has little effect on DNA G-quadruplexes, presumably due to unfavorable interactions with nucleobase and phosphate backbone SASAs exposed upon unfolding as well as the preferential hydration of the DNA G-quadruplex [23,28,30,37].

GB and L-proline are both protein stabilizers due to unfavorable interactions with (exclusion from) aliphatic carbon and amide oxygen SASAs exposed upon protein unfolding [39]. GB and L-proline destabilize DNA and RNA secondary structures [48,49,50], although GB has been shown to stabilize RNA tertiary structures depending on base sequence [48] and ionic strength [51]. The destabilization is driven by thermodynamically favorable interactions with nucleobase aromatic SASAs and less, but strong, unfavorable interactions with carbonyl and anionic phosphate oxygens [39]. These cosolutes preferentially destabilize GC-rich nucleic acid secondary structures to a greater extent than AT- or AU-rich duplexes because of the temperature-dependent nature of GB and L-proline interactions [49,50]. However, G-quadruplex stability has not been explored extensively in GB or L-proline solutions. While GB has been shown to stabilize G-quadruplex structures [30,32], it is unclear how the extent and type of SASA functional group exposure or burial drove stabilization.

In this work, we quantified the thermodynamic stability of the thrombin-binding aptamer G2 d(G_2_T_2_G_2_TGTG_2_T_2_G_2_) [9,10,11,52,53,54] in aqueous urea, GB, L-proline, and TMAO solutions containing potassium chloride using UV-absorbance spectroscopy. G2 is a well-known G-quadruplex that efficiently binds to thrombin, producing an anticoagulant effect [21]. Work has focused on producing G2 analogs with greater thrombin-binding efficiency [21,55]. G2 has also served as a model for understanding antibody-G-quadruplex interactions for detection of cellular G-quadruplexes [56]. G2 forms an antiparallel intramolecular G-quadruplex in a chair conformation with two stacked G-tetrads, two TT loops on the bottom and one TGT loop at the top of the structure [9,10]. We have also modified the nucleobase sequence of the loops to quantify loop contributions to G-quadruplex stability in cosolute solutions. Specifically, base substitutions in the TGT loop (TAT, TCT, TTT, UUU), the two TT loops (T_4_, U_2_), or in both loops (UGU and U_2_, UUU and U_2_) were the focus of our study. G2 and its base substitution variants have an SASA chemical landscape similar to DNA and RNA secondary and tertiary structures and therefore provide a test for a general mechanism of biopolymer stability in aqueous cosolute solutions.

## 2. Materials and Methods

### 2.1. Materials

The lyophilized DNA oligonucleotides (names in parentheses) d(G_2_T_2_G_2_TGTG_2_T_2_G_2_) (G2), d(G_2_T_2_G_2_TATG_2_T_2_G_2_) (G2-TAT), d(G_2_T_2_G_2_TCTG_2_T_2_G_2_) (G2-TCT), d(G_2_T_2_G_2_TTTG_2_T_2_G_2_) (G2-TTT), d(G_2_T_4_G_2_TGTG_2_T_4_G_2_) (G2-T4T4), d(G_2_U_2_G_2_TGTG_2_U_2_G_2_) (G2-U2U2), d(G_2_T_2_G_2_UUUG_2_T_2_G_2_) (G2-UUU), d(G_2_U_2_G_2_UGUG_2_U_2_G_2_) (G2-U), d(G_2_T_2_G_2_UGUG_2_T_2_G_2_) (G2-U2), and d(G_2_U_2_G_2_UUUG_2_U_2_G_2_) (G2-UUU-U2U2) were synthesized by Integrated DNA Technologies (IDT, Coralville, IA, USA) with standard desalting. The cosolutes glycine betaine (≥99%), L-proline (≥99%), trimethylamine *N*-oxide (≥98%), urea (ACS reagent grade) and buffer components HEPES (≥99.5%) and potassium chloride (ACS reagent) were purchased from Sigma-Aldrich (St. Louis, MO, USA). All reagents were used without further purification.

### 2.2. G-Quadruplex Preparation

The DNA oligonucleotides were solvated in a 10 mM HEPES, 100 mM potassium chloride pH 7.5 buffer to give stock concentrations ranging from 400 to 900 μM. DNA concentrations were determined by absorbance at 260 nm and 80 °C using the following molar extinction coefficients [10] in L mol^−1^ cm^−1^: 146,000 (G2), 147,000 (G2-TAT), 144,000 (G2-TCT), 141,000 (G2-TTT), 186,000 (G2-T4T4), 148,000 (G2-U2U2), 149,000 (G2-UUU), 156,000 (G2-U), 140,700 (G2-U2), and 158,000 (G2-UUU-U2U2). The extinction coefficient for G2-U2 was from IDT. DNA G-quadruplexes were annealed by heating to 90 °C, holding for 5 min, cooling to 60 °C, holding for fifteen minutes, cooling to 2 °C and holding for 40 min prior to storage at 4 °C.

DNA G-quadruplex-cosolute solutions for thermal denaturation were prepared gravimetrically by massing stock DNA G-quadruplex solution, HEPES buffer and a stock cosolute solution in HEPES buffer to ensure constant salt molality (m) and DNA concentration with adjustable cosolute molality. Cosolute concentrations ranged from 0.0 to 1.5 m in 0.25 m increments. Cosolute solution densities were measured using an Anton Paar DMA 5000 densitometer (Graz, Austria)to establish DNA G-quadruplex concentrations ranging from 25 to 45 μM. DNA G-quadruplex-cosolute solutions were prepared immediately prior to thermal denaturation.

### 2.3. G-Quadruplex Thermal Denaturation

DNA G-quadruplex thermal denaturation was monitored using a Cary 100 absorbance spectrophotometer (Agilent, Santa Clara, CA, USA) at 297 nm at a heating rate of 0.3 °C min^−1^. All solutions were degassed using a ThermoVac pump (MicroCal/Malvern, Worcestershire, UK) prior to thermal denaturation.

Absorbance (A) versus temperature (T) profiles were fit to a two-state melting model [57] given by

(1) $$A=\left(\alpha T+\beta \right)\cdot F(T)+(\gamma T+\delta )(1-F(T))$$

where α, β, γ, and δ were constants which described the baselines for the folded (α, β) and unfolded (γ, δ) regions and F(T) represented the fraction of folded G-quadruplex at temperature T (1−F(T) represented the fraction of unfolded G-quadruplex). F(T) was given by

(2) $$F\left(T\right)=\frac{1}{1+e^{\frac{-{∆H}_{obs}^{\circ }}{R}(\frac{1}{T}-\frac{1}{T_{m}})}}$$

where $∆H_{obs}^{\circ }$ was the observed van’t Hoff unfolding enthalpy, $T_{m}$ was the melting temperature at 50% folded G-quadruplex, and R was the ideal gas constant.

We quantified cosolute interactions with the solvent-accessible surface area exposed after unfolding the G-quadruplexes (ΔASA) using *m*-values. The *m*-value was defined by the dependence of the observed Gibbs energy difference $∆G_{obs}^{{}^{\circ}}$ between unfolded and folded G-quadruplex (or observed unfolding equilibrium constant $K_{obs}$) on cosolute molality, $m_{3}$ (Equation (3)) [38,58]

(3) $$m-value=\frac{\partial ∆G_{obs}^{{}^{\circ}}}{\partial m_{3}}=-RT\frac{\partial \ln K_{obs}}{\partial m_{3}}$$

The slope of a linear regression of the inverse of the melting temperature $T_{m}^{-1}$ as a function of *m*_3_ was used to calculate cosolute *m*-value/*RT* through Equation (4) [59]

(4) $$-\frac{∆H_{obs}^{\circ }}{R}\frac{\partial T_{m}^{-1}}{\partial m_{3}}=\frac{m-value}{RT}$$

$T_{m}$ values were averaged over duplicate trials at each cosolute concentration and the errors propagated to determine the standard error in *m*-value/*RT*.

### 2.4. ΔASA Calculations

The software tool *dr_sasa* (version 1) [60] was used to estimate ΔASA for the G2 G-quadruplex using a probe radius of 1.4 Å and the set of van der Waals radii from Chothia [61]. To estimate the solvent-accessible surface area of the folded G2 G-quadruplex, the surface area of every atom in each of the twelve G2 models in the Protein Data Bank entry 148D [62] were calculated and averaged. To estimate the solvent-accessible surface area of the unfolded G2 G-quadruplex, the average solvent-accessible surface areas of the atoms in guanine and thymine nucleobases in stacked (B-form) conformations were determined. The xleap module in AMBER 10 [63] was used to construct B-form DNA duplexes 5′-d(GCAAAGCAAACG)-3′ and 5′-d(GAAAGTAGAAAC)-3′. The solvent-accessible surface areas for each atom in the four single strands from the two duplexes were calculated. The 5′ guanine, 3′ guanine, and internal guanine and thymine nucleobase atom surface areas from each of the single strands were averaged. The surface area of unfolded G2 was then calculated by summing the average surface area of the guanine and thymine nucleobase atoms. ΔASA was finally calculated by subtracting the solvent-accessible surface area of folded G2 from that of unfolded G2.

## 3. Results

### 3.1. G-Quadruplex Thermal Unfolding

The unfolding molecularity of G2 G-quadruplex and its variants was determined from the dependence of the melting temperature $T_{m}$ on the G-quadruplex strand concentration. Melting temperatures were extracted by fitting thermal unfolding absorbance versus temperature curves using Equation (1) at strand concentrations ranging between 25 and 45 μM. The $T_{m}$ values had little dependence on strand concentration over this range (Figure S1—Supplementary Materials). All G-quadruplexes unfolded in a unimolecular fashion and therefore existed as intramolecular complexes in agreement with previous studies [10]. Thereafter, all thermal unfolding experiments in aqueous cosolute solutions were conducted at a 25 μM G-quadruplex strand concentration. Given the pKa of HEPES at 25 °C and its reduction rate with temperature, we estimated a pH between 6.9 and 7.0 at 63.5 °C, the highest denaturation temperature seen in our studies [64]. DNA begins to protonate below pH 5, so we did not expect any pH-induced denaturation [65].

**Figure 1:**
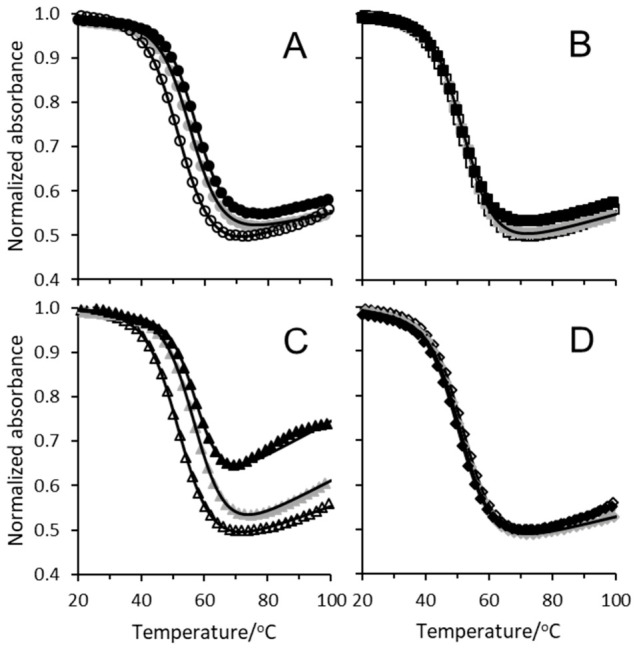
Thermal denaturation of G2 G-quadruplex in aqueous cosolute solutions. Normalized absorbance as a function of temperature for 25 μM G2 in (**A**) glycine betaine, (**B**) proline, (**C**) TMAO, and (**D**) urea solutions with 10 mM HEPES, 100 mM potassium chloride at pH 7.5. Solutions of 0 m cosolute (open marks), 0.75 m cosolute (gray marks), and 1.5 m cosolute (filled marks). Nonlinear fits to data using Equation (1) shown as solid lines.

Figure 1 shows representative absorbance versus temperature unfolding profiles for the G2 G-quadruplex in aqueous GB, proline, TMAO, and urea solutions at 0, 0.75, and 1.5 m. Both GB and TMAO robustly stabilized the G2 G-quadruplex, shifting the unfolding curves to higher temperatures with increasing the concentration of cosolute. Previous work has shown the stabilizing effects of TMAO on G-quadruplex thermal stability [30,37] while GB has been shown to stabilize RNA tertiary folds, although this stabilizing effect was salt- and sequence-dependent [48,51]. The stabilizing effects of GB and TMAO on G2 were substantial, as the unfolding curves (Figure 1) shifted upward by nearly 6 °C at 1.5 m cosolute relative to cosolute-free solution. This behavior was in contrast to the weaker stabilization by TMAO [66,67] and the destabilization by GB of double-stranded DNA and RNA [49,68].

In contrast to GB and TMAO, there was minor destabilization (less than 1 °C decrease in $T_{m}$) of the G2 G-quadruplex with proline and urea (Figure 1). While urea has been shown to stabilize G-quadruplexes with single-nucleobase loops [13], urea generally destabilizes G-quadruplex structures [30,37] as observed in this study. Both urea and proline are prolific destabilizers of double-stranded DNA, double-stranded RNA, and RNA tertiary structures [48,50,51,69], lowering melting temperatures by 5 °C or more at 1.5–2 m cosolute.

Nonlinear regression of Equation (1) to the data in Figure 1 is shown as solid lines and was used to extract the observed van’t Hoff unfolding enthalpy $∆H_{obs}^{\circ }$ and $T_{m}$. Tables S1–S4 (Supplementary Materials) contain the average $∆H_{obs}^{\circ }$ for G2 and all its variants at all concentrations of cosolutes used in this study. Average $∆H_{obs}^{\circ }$ values in cosolute-free solutions were in general agreement with previous work [10]. The only discrepancies were G2-T4T4 ($∆H_{obs}^{\circ }$ in this study was approximately 10 kcal mol^−1^ larger), G2-UUU ($∆H_{obs}^{\circ }$ in this study was approximately 6 kcal mol^−1^ larger), and G2-U ($∆H_{obs}^{\circ }$ in this study was approximately 13 kcal mol^−1^ larger). Reference [10] did not include G2-U2 so we had no comparison for this G2 variant.

In general, glycine betaine, proline, and urea increased $∆H_{obs}^{\circ }$ by approximately 2–15% at the largest cosolute concentration (1.5 m), whereas TMAO produced larger increases in $∆H_{obs}^{\circ }$ of 8–30% dependent on nucleobase sequence. The large increase in $∆H_{obs}^{\circ }$ in TMAO was consistent with the work of Ueda et al. on an intramolecular G-quadruplex, although $∆H_{obs}^{\circ }$ decreased with urea concentration [30]. However, Tariq and co-workers demonstrated that changes in $∆H_{obs}^{\circ }$ with urea concentration depended on the G-quadruplex nucleobase sequence and even high concentrations of urea eventually attenuated $∆H_{obs}^{\circ }$ [13]. While there are few data on G-quadruplex unfolding in GB and proline solutions, the observed increases in $∆H_{obs}^{\circ }$ in this study were similar in magnitude to those observed in double-stranded RNA [50].

The circular dichroism (CD) spectra of G2 and its variants show a positive band centered at 292 nm, corresponding to a “chair” conformation in cosolute-free solutions [10]. In aqueous cosolute solutions, we found very similar hyperchromicities for the G-quadruplexes compared to cosolute-free solutions even at the highest concentration of cosolutes, except for TMAO (Figure 1). The smaller hyperchromicities at 1.25 and 1.5 m TMAO were potentially due to the single strands of the unfolded G-quadruplexes favoring more compact structures or different hydration levels of the unfolded G-quadruplex strands in high concentrations of TMAO [28,70]. This suggested that the conformations of the G-quadruplexes used in this study were not overly perturbed by the presence of cosolute. Indeed, Ueda et al. found that TMAO and urea did not largely affect the conformation of an intramolecular G-quadruplex, even at the highest concentrations of TMAO and urea used in their study [30]. The increase in $∆H_{obs}^{\circ }$ with cosolute concentration reflected a net increase in attractive interactions in the folded, native state or a decrease in attractive interactions in the unfolded state. However, we had no information on the enthalpies of folded or unfolded G-quadruplexes in cosolute solutions relative to cosolute-free solutions. We hypothesize that an increase in $∆H_{obs}^{\circ }$ with cosolute could be accounted for by changes in the amounts of water, ion, or cosolute accumulation at the folded G-quadruplex surface or fewer water interactions with the unfolded G-quadruplex surface.

The average $T_{m}$ values for the G-quadruplexes at each cosolute concentration are tabulated in Tables S5–S8 (Supplementary Materials). The average $T_{m}$ values in cosolute-free solutions were in excellent agreement with previous studies [10]. In GB and TMAO solutions, G-quadruplexes were substantially stabilized and the magnitude of stabilization was only weakly dependent on nucleobase sequence. In general, the average $T_{m}$ values increased by 7–8 °C in 1.5 m TMAO while they increased by 6–7 °C in 1.5 m GB relative to the cosolute-free solution. Our results agreed with previous work showing that TMAO demonstrated the ability to significantly stabilize intramolecular G-quadruplexes [30,37]. An increase in $T_{m}$ suggested more favorable interactions of GB and TMAO with the folded G-quadruplex than with the newly exposed surface area after unfolding of the G-quadruplexes.

Proline weakly stabilized or destabilized G-quadruplexes dependent on nucleobase sequence as indicated by an increase or decrease in average $T_{m}$ values of 1–2 °C at 1.5 m (Table S6). Replacement of the thymine nucleobase with uracil in the TGT loop resulted in a slight decrease in $T_{m}$ values with proline concentration. However, replacement of thymine with uracil in the T_2_ loops increased $T_{m}$ slightly with proline concentration and even offset the reduction of $T_{m}$ after modification of the TGT loop with uracil (see G2-UUU-U2U2 in Table S6). While replacement of G in the TGT loop with A, C, or T led to destabilization of the entire G-quadruplex in cosolute-free solutions, there was a minor increase in $T_{m}$ with proline concentration. Regardless, the small effect of proline on $T_{m}$ values suggested weak net-favorable or unfavorable sequence-dependent proline interactions with the newly exposed G-quadruplex surface area after unfolding.

Urea generally decreased G-quadruplex $T_{m}$ values by 1–2 °C at 1.5 m (Table S8), which agreed with thermal unfolding studies with different G-quadruplexes [13,27,30]. Urea had weak net-favorable interactions with the newly exposed G-quadruplex surface area after unfolding. However, like proline, replacement of thymine with uracil in the T_2_ loops resulted in a minor $T_{m}$ increase with urea.

Entropies of unfolding increased with cosolute concentration, regardless of if the cosolute was a stabilizer or destabilizer (Tables S9–S12). Cosolute interactions with the G-quadruplex folded and unfolded surface areas resulted in differential water, ion, or cosolute release to ensure unfolding entropies increased with cosolute molality for nearly all cosolute and G-quadruplex combinations. Previous work illustrated a similar trend with GB, proline, and urea with DNA and RNA duplexes and an RNA tertiary fold [49,50,66].

### 3.2. Cosolute m-Value/RT Values

**Figure 2:**
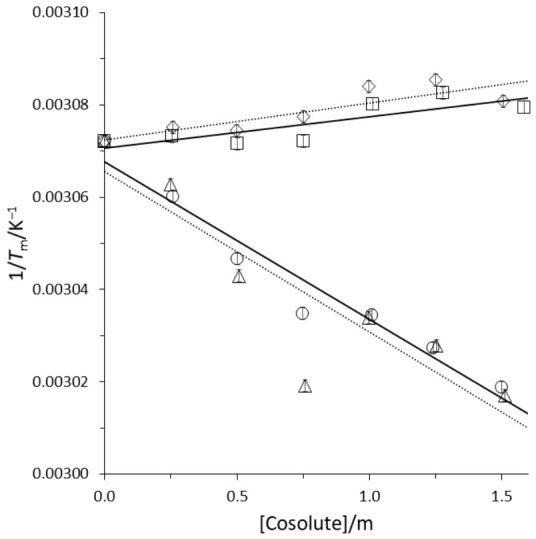
G2 inverse melting temperature $T_{m}^{-1}$ as a function of cosolute molality. Glycine betaine (circles), proline (squares), TMAO (triangles), and urea (diamonds) in 10 mM HEPES, 100 mM potassium chloride buffer at pH 7.5. Solid linear regression lines (glycine betaine, proline), dashed linear regression lines (TMAO, urea). Error bars approximately the size of the markers.

Using the $T_{m}$ values from Tables S5–S8, linear regression of ${T_{m}}^{-1}$ as a function of cosolute molality yielded the slope necessary to determine the cosolute *m*-value/*RT* (Equation (4)) for each of the G-quadruplexes used in this study. A representative plot of ${T_{m}}^{-1}$ as a function of GB, proline, TMAO, and urea molality is shown in Figure 2 for the G2 quadruplex. There was no evidence of a higher-order dependence of ${T_{m}}^{-1}$ on cosolute molality than a linear dependence over 0–1.5 m cosolute for each of the G-quadruplexes. Cosolute *m*-value/*RT* for each G-quadruplex was calculated using $∆H_{obs}^{\circ }$ in cosolute-free solutions from Tables S1–S4.

**Figure 3:**
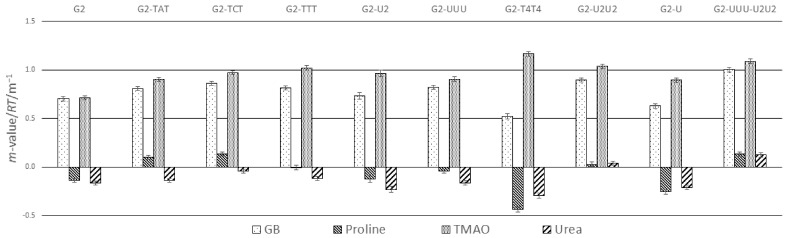
*m*-value/*RT* values for G2 and variant G-quadruplexes in glycine betaine (GB), proline, TMAO, and urea solutions. Melting temperature ranges used to determine *m*-value/*RT*: G2 (52.4–58.3 °C), G2-TAT (46.1–53.4 °C), G2-TCT (43.6–51.7 °C), G2-TTT (47.7–55.7 °C), G2-U2 (56.1–62.9 °C), G2-UUU (47.7–54.8 °C), G2-T4T4 (41.6–49.2 °C), G2-U2U2 (52.9–60.7 °C), G2-U (57.4–63.3 °C), G2-UUU-U2U2 (48.1–55.7 °C).

Figure 3 displays the *m*-value/*RT* values for each G-quadruplex in GB, proline, TMAO, and urea solutions. Based on calorimetric enthalpy data [10], all G2 variants unfolded in two-state transitions with no intermediates in the unfolding pathway. Only G2 exhibited non-two-state behavior which was hypothesized to be due to end-to-end G-quadruplex dimers. For our calculations of cosolute *m*-value/*RT* values, $∆H_{obs}^{\circ }$ values in cosolute-free solutions from this study were used due to the general agreement with those in [10]. Therefore, our calculated cosolute *m*-value/*RT* for G2 might have overestimated its true *m*-value/*RT*. GB and proline interactions with nucleic acid surfaces are temperature-dependent [49,50]. Therefore, the range of $T_{m}$ values used to determine the *m*-value/*RT* values are also given in Figure 3. Except for G2-TCT, G2-U, and G2-U2, all G-quadruplex *m*-value/*RT* values were determined in $T_{m}$ ranges that have overlap. However, since the $T_{m}$ ranges for G2-TCT, G2-U, and G2-U2 were not grossly disparate from the other G-quadruplexes and overlap for a few in this set, we feel confident including their *m*-value/*RT* values in our analysis.

Positive *m*-value/*RT* values in Figure 3 indicated stabilization of G-quadruplexes, reflected as an increase in $T_{m}$ or the free energy of unfolding or a decrease in the observed unfolding equilibrium constant with cosolute concentration (Equations (3) and (4)). Negative *m*-value/*RT* values indicated a decrease in G-quadruplex stability. The magnitude of stabilization of all the G-quadruplexes by GB and TMAO was significantly larger than stabilization or destabilization by proline or urea. The magnitude of *m*-value/*RT* is proportional to both the magnitude of ΔASA exposed upon unfolding and the strength of cosolute interactions with chemical functional groups comprising that surface area [39,71]. For the G-quadruplexes in Figure 3, negative (positive) *m*-value/*RT* values indicated net-favorable (unfavorable) interactions of cosolute with the ΔASA exposed during unfolding.

GB has demonstrated unfavorable interactions with nucleobase and phosphate oxygen solvent-accessible surfaces, but favorable interactions with nucleobase aromatic carbon and nitrogen surfaces [39]. TMAO demonstrated unfavorable interactions with all chemical functional groups except cationic nitrogen (amine) surfaces [71]. Therefore, it was not surprising to observe that TMAO was a stronger stabilizer (*m*-value/*RT* > 0) for any given G-quadruplex compared to GB due to its greater net-unfavorable interactions with ΔASA (Figure 3).

Proline has unfavorable interactions with aliphatic, nucleobase and phosphate oxygen surfaces, but favorable interactions with cationic nitrogen and aromatic carbon and nitrogen surfaces [39]. Urea has favorable interactions with all surface area types except for minor unfavorable interaction with cationic nitrogens [39]. Predictably, urea was mostly a net destabilizer of the G-quadruplexes (*m*-value/*RT* < 0), but proline fluctuated between a G-quadruplex stabilizer and destabilizer dependent on ΔASA chemical composition and magnitude (Figure 3).

Replacement of G by A, C, or T in the TGT loop led to less destabilization by proline and urea and nearly the same or greater stabilization by GB and TMAO (Figure 3). There was no clear trend when comparing G2 with G2-U2, G2-UUU, G2-U2U2, and G2-U G-quadruplexes with thymine replaced by the uracil nucleobase. Since proline has unfavorable interactions with aliphatic carbons, a reasonable assumption might expect less favorable interaction with thymine and its methyl group and therefore more favorable interaction with ΔASA for uracil-containing G-quadruplexes. However, only in the case of G2-U was there a greater destabilization by proline compared to G2. The addition of more thymine nucleobases in the lower loops of G2-T4T4 led to greater destabilization, possibly due to larger ΔASA. In the case of urea, with its weak favorable interaction with aliphatic carbons, replacement of thymine with uracil nucleobases did not translate to less destabilization.

The conformation of the average folded structures of the G-quadruplexes in aqueous solution besides G2 was unknown. Any small differences in magnitude and chemical composition of ΔASA between the G-quadruplexes were thus unpredictable. However, the enthalpy contribution of the loops to G-quadruplex unfolding indicated that base-stacking occurs within the loops and loop nucleobase stacking can occur on the G-quartets [10]. This behavior was observed in the NMR-derived structure of G2 with guanine of the TGT loop stacked on the G-quartets [62]. Stacking in the G2 TGT loop led to the thymine methyl groups exposed to solvent while the thymine methyl groups in the T2 loops were in close proximity, potentially minimizing interaction with solvent. The enthalpic contribution to loop interactions in G2 variants was larger than those in G2 alone, which suggested stronger stacking in loops or on the G-quartets [10]. Loop unfolding enthalpies also included effects from water uptake, potassium ion release and cosolute exchange. Accordingly, we propose that the differences in *m*-value/*RT* arose from different loop folds and loop nucleobase stacking on the G-quartets with slightly different magnitudes of ΔASA and chemical functional groups exposed.

## 4. Discussion

### 4.1. G-Quadruplex-Cosolute m-Value Contributions

For comparison to cosolute *m*-values from other nucleic acids, we determined GB, proline, TMAO, and urea *m*-values at the G-quadruplex $T_{m}$ values in cosolute-free solutions (Tables S5–S8). Since the *m*-value is proportional to ΔASA, we normalized the *m*-value for the G2 quadruplex with its estimated ΔASA of 621 Å^2^ (see Materials and Methods Section 2.4). We only determined G2 ΔASA as it was the only G-quadruplex in our set with an NMR-derived structure in aqueous solution [62]. Using G2 *m*-value/*RT* values from Figure 3, we determined ΔASA-normalized values of 3.1 ± 0.1, −0.60 ± 0.09, 3.1 ± 0.1, and −0.72 ± 0.09 cal mol^−1^ m^−1^ Å^−2^ for GB, proline, TMAO, and urea, respectively.

Table 1 contains average urea ΔASA-normalized *m*-values for the G2 quadruplex and a variety of DNA and RNA structures from other sources [27,69,72,73]. In general, average urea ΔASA-normalized *m*-values were similar regardless of DNA or RNA structure. The average urea ΔASA-normalized *m*-value from Aslanyan et al. was about two-thirds the magnitude for that of G2, although the Tel22 G-quadruplex used sodium ions in the G-quadruplex cavity [27] compared to potassium ion in this study. The average urea ΔASA-normalized *m*-value from Shelton and co-workers for a set of RNA duplexes ranging in size from a hexamer to a octadecamer in length as well as yeast tRNA^Phe^ was the smallest in magnitude [72]. However, they assumed maximally exposed nucleobases in ΔASA. The similarity in the magnitude of average urea ΔASA-normalized *m*-values between different nucleic acid structures underscores that urea possesses similar favorable interaction potentials with nearly all nucleic acid surface types exposed in ΔASA [69].

Like urea, proline destabilized folded nucleic acid structures. Table 1 contains the average ΔASA-normalized proline *m*-value for G2 and a series of RNA dodecamers with melting temperatures similar to those for the G-quadruplexes used in this study [50]. Differences in proline *m*-values in Table 1 reflected different surface area types exposed after unfolding G2 and RNA duplexes because proline has favorable interactions with some surface area types and unfavorable interactions with others [39].

Unsurprisingly, the average ΔASA-normalized GB *m*-values differed significantly between a series of RNA dodecamers and G2. Like proline, GB has favorable interactions with essentially only nucleobase ΔASA and unfavorable interactions with all other surface area types (albeit with different interaction potential magnitude than proline) [39]. Thus, the different ΔASA composition between the RNA duplexes and G2 drove different degrees of GB interaction. TMAO stabilized DNA and RNA structures (Table 1). Using the average ΔASA for the DNA dodecamers in reference [69], we estimated a ΔASA-normalized TMAO *m*-value of 0.93 ± 0.01 cal mol^−1^ M^−1^ Å^−2^ for an eight-nucleobase-pair DNA oligomer [66]. Using the ΔASA values from [73] for four different RNA secondary and tertiary structures assuming A-form RNA single strands, we determined an average ΔASA-normalized TMAO *m*-value of 0.97 ± 0.29 cal mol^−1^ m^−1^ Å^−2^, the maximum value in this average being 1.9 cal mol^−1^ m^−1^ Å^−2^ for the tetraloop receptor RNA [74]. Since TMAO has favorable interactions with only cationic amine surface areas [71], we might have expected similar average ΔASA-normalized TMAO *m*-values across all DNA and RNA structures like that for urea. However, TMAO interaction potentials are much larger in magnitude and differ more between different surface area types compared to urea [71]. Therefore, differences in G2, DNA duplex, and RNA structure ΔASA chemical composition must have played a critical role in determining the average ΔASA-normalized TMAO *m*-values.

### 4.2. Predicted G2 m-Value/RT G-Quartet and Loop Contributions

In an effort to gain more insight into the behavior of G2 and its variants in GB, proline, TMAO and urea solutions, we predicted the G2 *m*-value/*RT* using cosolute α-value interaction parameters [39,71]. The α-value represented the surface area normalized *m*-value of a particular functional group *i*. *m*-value/*RT* values were calculated by summing the α_i_-values weighted by the change in solvent-accessible surface area ΔASA_i_ of surface type *i:*

(5) $$\frac{m-value}{RT}=\sum_{i}\alpha_{i}∆{ASA}_{i}$$

In Equation (5), the exposure of a single potassium ion sandwiched within the G-quartets was accounted for, except for TMAO, since its interaction with potassium was weak [74]. The α_i_-values from references [39,71] were specific to aliphatic carbon, aromatic carbon, hydroxyl oxygen, amide oxygen, amide nitrogen, carboxylate oxygen, phosphate oxygen, and cationic nitrogen. We made the following assumptions used in [50] to apply Equation (5) to the G2 quadruplex: (1) the α_i_-values for phosphorus, O4′, and O5′ were assigned values of zero because of their small ΔASA_i_ values and no corresponding α_i_-values for these surface types, (2) nitrogen atoms in the nucleobase rings were treated as aromatic carbons except for N1 on guanine and N3 on uracil and thymine which were treated as amide nitrogens (with a similar α_i_-value to aromatic carbon), (3) primary amines on nucleobases were treated as amide nitrogens (not cationic nitrogen) due to the ability to donate and accept hydrogen bonds (amide and cationic nitrogen have similar α_i_-values), (4) O6 on guanine and O2 and O4 on uracil were treated as amide oxygen, (5) O2 on cytosine was treated as amide oxygen because it does not qualify as a carboxylate oxygen. In the case of TMAO, phosphate oxygens were treated as carboxylate oxygen since no anionic oxygen α_i_-value was given in reference [71].

**Figure 4:**
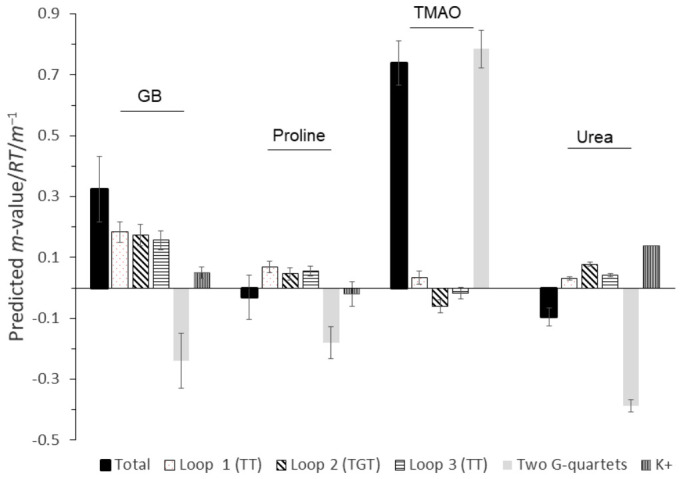
Predicted *m*-value/*RT* values for the G2 quadruplex in glycine betaine (GB), proline, TMAO, and urea solutions. The total *m*-value/*RT* value represents cosolute interactions with the entire new surface area exposed after G2 unfolding (ΔASA). Contributions to total *m*-value/*RT* values were parsed into loop 1 (two thymine nucleotides closest to the 5′ end of G2), loop 2, loop 3, the two G-quartets, and potassium cation contributions. The TMAO-potassium cation contribution was not included since this value was considered negligible.

Figure 4 plots the predicted GB, proline, TMAO, and urea *m*-value/*RT* values for G2 including *m*-value/*RT* contributions from the loops, G-quartets, and potassium ions. While we did not have exact agreement with the magnitude of experimentally determined *m*-value/*RT* values in Figure 3, we successfully predicted the sign of *m*-value/*RT*. Thus, we predicted the small destabilizing effect of proline and urea and the larger stabilizing effect of GB and TMAO on the G2 G-quadruplex. We did not expect exact agreement because the interaction potentials were typically measured in salt-free solutions. Except for TMAO, exposure of G-quartet ΔASA had negative *m*-value/*RT* values. Exposure of the G-quartet ΔASA included significant aromatic surface area and favorable interactions with GB, proline, and urea, but unfavorable interactions with TMAO. Exposure of the potassium ion within G2 contributed little in the case of GB, proline, and urea.

With proline and urea, predicted unfavorable interactions in the loop ΔASA nearly offset the favorable interactions with the G-quartets, while unfavorable loop interactions with GB overwhelmed the favorable G-quartet interactions. GB and proline interactions with loop ΔASA included increased unfavorable interactions due to the net exposure of phosphate oxygen surface area and a loss of favorable interactions with nucleobase surface area due to the net burial of nucleobases in the unfolded structure. For urea, the net loss of favorable interactions with nucleobase surface area was sufficient to predict unfavorable interactions with loop ΔASA.

Our ΔASA calculations for G2 unfolding indicated the total surface area exposed was ~620 Å^2^, assuming the unfolded G2 sequence was in a B-form conformation. This resulted in an estimated burial of 44 Å^2^ of aliphatic and aromatic carbon surface area (−44 Å^2^ contribution to ΔASA). One of the driving forces of kosmotrope stabilization of proteins with cosolutes such as GB, proline, and TMAO [75] is strengthening the hydrophobic effect by ordering bulk water. If a similar mechanism existed for G2, then based on our ΔASA calculations, this contribution would be negligible (or even slightly destabilizing) despite very strong stabilization of G2 by GB and TMAO compared to other nucleic acid structures (Table 1). Proline’s behavior as a weak destabilizer of G2 was opposite to the action of GB and TMAO while also being classified as a protein kosmotrope. In addition, urea’s assignment as a protein chaotrope would suggest its disordering of bulk water would have had little or even a slightly stabilizing effect on G2 structure based on our ΔASA calculations [76]. However, urea’s ΔASA-normalized *m*-value for G2 was comparable to a host of other DNA and RNA structures (Table 1). Our results indicated the use of kosmotropic or chaotropic cosolute descriptors may not fully capture a cosolute’s ability as a G-quadruplex stabilizer or destabilizer [23,76,77].

We propose that the differential stabilities of G2 and its variants were driven by strong favorable interactions of GB, proline, and urea with G-quartet ΔASA modulated by unfavorable interactions with loop ΔASA. TMAO stabilization was determined mostly by unfavorable interactions with G-quartet nucleobase ΔASA.

## 5. Conclusions

We quantified the effects of the cosolutes GB, proline, TMAO, and urea on the thermodynamic stability of the thrombin-binding aptamer G2 and its variants with loop nucleobase substitutions. Our studies revealed the following:

(1) G2 and variant G-quadruplex unfolding enthalpies increased with cosolute molality with the largest increase at 1.5 molal TMAO. This reflected a larger loss of solvent, cosolute, and G-quadruplex interactions in cosolute solutions relative to cosolute-free solutions. Unfolding entropies were positive for all G-quadruplexes and increased with cosolute molality for all G-quadruplexes, presumably due to differential water, ion, or cosolute release upon unfolding.
(2) GB and TMAO significantly stabilized the G-quadruplexes in this study, increasing the melting temperature by 6–8 °C at 1.5 m. GB and TMAO *m*-value/*RT* values were dependent on loop sequence, but were large and positive (0.5 m^−1^ or larger). This stabilization resulted from net-unfavorable interactions with the surface area exposed after unfolding. GB and TMAO have net-favorable interactions with aromatic and cationic nitrogen surface areas, respectively, and unfavorable interactions with all other surface area types. This suggested exposure of different chemical functional groups in the newly exposed surface area landscape contributed to G-quadruplex stabilization in GB solutions.
(3) Proline acted as a weak G-quadruplex stabilizer or destabilizer (an increase or decrease in melting temperature of 1–2 °C at 1.5 m) depending on loop sequence. Proline *m*-value/*RT* values were therefore small in magnitude. Proline has favorable interactions with aromatic and nitrogen surface areas and the degree of exposure in the unfolded surface area modulated proline’s ability as a stabilizer or destabilizer. Urea acted as a general weak G-quadruplex destabilizer since it has favorable interactions with nearly all surface area types exposed during unfolding.
(4) G2 GB and TMAO *m*-values normalized by the predicted change in solvent-accessible surface area after unfolding are much larger than other DNA and RNA structures from the literature. This difference reflected different chemical landscapes in the solvent-accessible surface areas exposed after unfolding G2 and DNA and RNA folded structures. G2 urea *m*-values normalized by the G2 surface area exposed after unfolding are in good agreement with other DNA and RNA structures, presumably due to the general favorable interactions of urea with most chemical functional groups. G2 proline *m*-values after normalization are in general agreement with other RNA and DNA structures, but there appeared to be more dependence on the chemical makeup of the G2 surface area exposed after unfolding.
(5) Our results suggested that cosolute-induced stabilization or destabilization of G2 and its variants arose primarily from strong favorable interactions of GB, proline, and urea with G-quartet surface area exposed after unfolding attenuated by unfavorable interactions with loop newly exposed surface area. TMAO stabilization was determined mostly by unfavorable interactions with G-quartet nucleobase surface area exposed after unfolding.

## Figures and Tables

**Table 1:** Average ΔASA-normalized *m*-value (cal mol^−1^ m^−1^ Å^−2^) for DNA and RNA structures.

	Glycine Betaine	Proline	TMAO	Urea
G2 quadruplex (this work)	3.1 ± 0.1	−0.60 ± 0.09	3.1 ± 0.1	−0.72 ± 0.09
Tel22 quadruplex [27] ^a^				−0.46 ± 0.07
DNA duplexes (12 bp) [69]				−0.65 ± 0.11
DNA duplex (8 bp) [66] ^a^			0.93 ± 0.01	
RNAs (6 to 18 bp, tRNA^Phe^) [72] ^a,b^				−0.099 ± 0.004
RNA duplexes (12 bp) [49,50,69]	−0.63 ± 0.10	−1.1 ± 0.1		−0.70 ± 0.06
RNA hairpin, tar-tar *, A-riboswitch, tetraloop-receptor motif [73,74]			0.97 ± 0.29	−0.70 ± 0.22

^a^ *m*-value units are cal mol^−1^ M^−1^ Å^−2^. ^b^ Assuming maximally exposed nucleobases in ΔASA. tar-tar *: a RNA complex in refereces [73,74].

## Data Availability

Data from this work has been deposited in the Mendeley Data communal data repository with accession DOI: 10.17632/rj6j66h835.1.

## Supplementary Materials

The following supporting information can be downloaded at https://www.mdpi.com/article/10.3390/biom16050697/s1, Figure S1: Average quadruplex melting temperature *T*_m_ as a function of quadruplex strand concentration; Table S1: Observed unfolding enthalpies $∆H_{obs}^{\circ }$ (kcal mol^−1^) assuming a two-state model for G2 quadruplex variants in aqueous glycine betaine (GB) solutions; Table S2: Observed unfolding enthalpies $∆H_{obs}^{\circ }$ (kcal mol^−1^) assuming a two-state model for G2 quadruplex variants in aqueous proline solutions; Table S3: Observed unfolding enthalpies $∆H_{obs}^{\circ }$ (kcal mol^−1^) assuming a two-state model for G2 quadruplex variants in aqueous TMAO solutions; Table S4: Observed unfolding enthalpies $∆H_{obs}^{\circ }$ (kcal mol^−1^) assuming a two-state model for G2 quadruplex variants in aqueous urea solutions; Table S5: Transition temperatures *T*_m_ (°C) assuming a two-state model for G2 quadruplex variants in aqueous glycine betaine (GB) solutions; Table S6: Transition temperatures *T*_m_ (°C) assuming a two-state model for G2 quadruplex variants in aqueous proline solutions; Table S7: Transition temperatures *T*_m_ (°C) assuming a two-state model for G2 quadruplex variants in aqueous TMAO solutions; Table S8: Transition temperatures *T*_m_ (°C) assuming a two-state model for G2 quadruplex variants in aqueous urea solutions; Table S9: Observed unfolding entropies $\Delta S_{\mathrm{obs}}^{\circ }$ (cal mol^−1^ K^−1^) assuming a two-state model for G2 quadruplex variants in aqueous glycine betaine (GB) solutions; Table S10: Observed unfolding entropies $\Delta S_{\mathrm{obs}}^{\circ }$ (cal mol^−1^ K^−1^) assuming a two-state model for G2 quadruplex variants in aqueous proline solutions; Table S11: Observed unfolding entropies $\Delta S_{\mathrm{obs}}^{\circ }$ (cal mol^−1^ K^−1^) assuming a two-state model for G2 quadruplex variants in aqueous TMAO solutions; Table S12: Observed unfolding entropies $\Delta S_{\mathrm{obs}}^{\circ }$ (cal mol^−1^ K^−1^) assuming a two-state model for G2 quadruplex variants in aqueous urea solutions.

## Abbreviations

The following abbreviations are used in this manuscript:

GB	Glycine betaine
TMAO	Trimethylamine N-oxide
SASA	Solvent-accessible surface area
DNA	Deoxyribonucleic acid
RNA	Ribonucleic acid
T	Thymine
G	Guanine
A	Adenine
C	Cytosine
U	Uracil
ΔASA	Solvent-accessible surface area exposed after unfolding
CD	Circular dichroism
bp	Nucleobase pair
