# Supplementary material for: Effects of Loop Nucleobase Substitution on G-Quadruplex Thermal Stability in Aqueous Glycine Betaine, Proline, TMAO, and Urea Solutions
Source: Biomolecules. 2026 May 8;16(5):697. doi: 10.3390/biom16050697 (PMC13204723; doi:10.3390/biom16050697)
Supplement: Supplementary file 1 [file biomolecules-16-00697-s001.zip › biomolecules-4267574-supplementary.pdf]

Effects of Loop Nucleobase Substitution on G-quadruplex Thermal Stability in Aqueous Glycine Betaine, Proline, TMAO, and Urea Solutions

Jeffrey J. Schwinefus , Marija Corluka, Isabella Dobrinski, Stella L. Jaeckle, Joshua Kim, Grace Knowlan,

Hannah Omodt, Noah Otto, Mari V. Reid, Reid Rognerud and Kathryn M. Stein

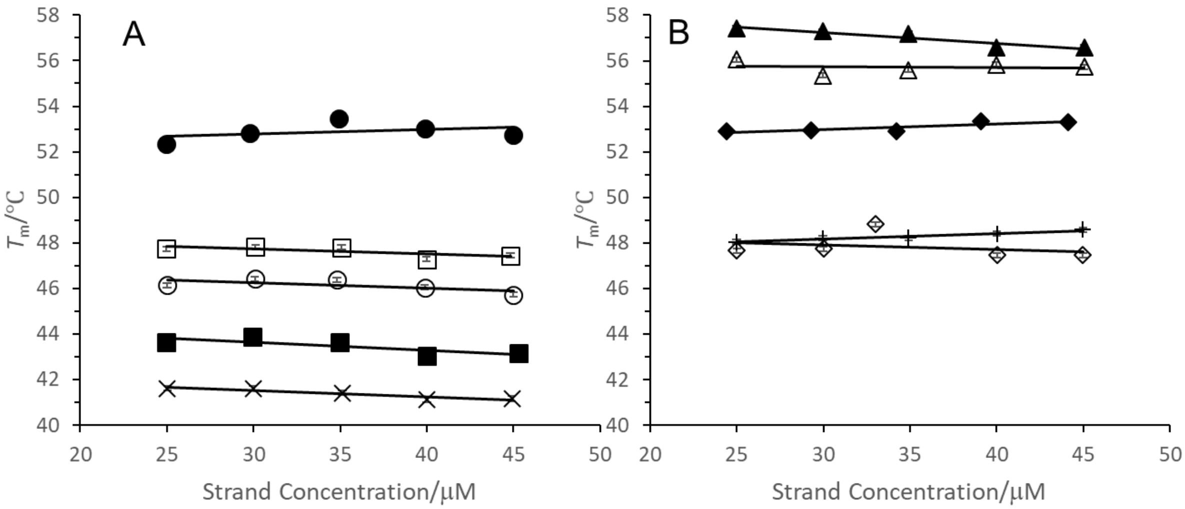

Figure S1: Average quadruplex melting temperature T_m as a function of quadruplex strand concentration. Data points are averages of two trials, error bars the size or smaller than markers. A) G2 (●), G2-TAT (○), G2-TCT (■), G2-TTT (□), G2-T4T4 (×), B) G2-U2U2 (♦), G2-UUU (◊), G2-U (▲), G2-U2 (△), G2-UUU-U2U2 (+).

Table S1: Observed Unfolding Enthalpies ΔH°_{obs} (kcal mol^{-1})[a] Assuming a Two-State Model for G2 Quadruplex Variants in Aqueous Glycine Betaine (GB) Solutions[b]

GB/mol kg^{-1}	G2	G2-TAT	G2-TCT	G2-TTT	G2-T4T4	G2-U2U2	G2-UUU	G2-U	G2-U2	G2-UUU-U2U2
0.00	41.1 ± 0.2	38.2 ± 0.2	38.8 ± 0.1	41.8 ± 0.2	49.3 ± 0.3	44.1 ± 0.3	43.0 ± 0.1	48.2 ± 0.4	46.1 ± 0.6	46.1 ± 0.5
0.25	42.4 ± 0.1	39.0 ± 0.5	40.1 ± 0.1	42.4 ± 0.1	49.6 ± 0.1	45.2 ± 0.1	42.9 ± 0.1	48.5 ± 0.2	45.8 ± 0.1	46.5 ± 0.2
0.50	43.2 ± 0.1	40.1 ± 0.1	40.7 ± 0.1	43.4 ± 0.1	49.7 ± 0.2	46.2 ± 0.1	44.2 ± 0.1	48.2 ± 0.6	49.1 ± 0.4	48.1 ± 0.3
0.75	44.1 ± 0.2	41.1 ± 0.1	41.5 ± 0.2	44.0 ± 0.2	50.0 ± 0.2	46.7 ± 0.2	45.3 ± 0.1	51.7 ± 0.3	48.2 ± 0.1	49.3 ± 0.3
1.00	47.4 ± 0.2	42.7 ± 0.1	42.9 ± 0.1	45.7 ± 0.1	50.5 ± 0.2	48.4 ± 0.1	45.8 ± 0.1	52.2 ± 0.2	49.2 ± 0.1	49.4 ± 0.2
1.25	46.3 ± 0.1	43.3 ± 0.1	43.9 ± 0.1	46.3 ± 0.1	50.7 ± 0.2	49.1 ± 0.2	46.3 ± 0.1	52.7 ± 0.2	49.6 ± 0.1	50.4 ± 0.3
1.50	46.3 ± 0.1	43.1 ± 0.2	44.5 ± 0.2	46.9 ± 0.2	50.4 ± 0.2	49.6 ± 0.2	46.6 ± 0.1	53.4 ± 0.2	49.8 ± 0.2	50.3 ± 0.2

[a]Values are averages of two trials at 25 µM DNA except at 0 m cosolute which are averaged over 25 – 45 µM DNA; nonlinear regression uncertainties used for average value error propagation
[b]10 mM HEPES, 100 mmolal potassium chloride

Table S2: Observed Unfolding Enthalpies ΔH°_{obs} (kcal mol^{-1})[a] Assuming a Two-State Model for G2 Quadruplex Variants in Aqueous Proline Solutions[b]

Proline/mol kg^{-1}	G2	G2-TAT	G2-TCT	G2-TTT	G2-T4T4	G2-U2U2	G2-UUU	G2-U	G2-U2	G2-UUU-U2U2
0.00	41.1 ± 0.2	38.2 ± 0.2	38.8 ± 0.1	41.8 ± 0.2	49.3 ± 0.3	44.1 ± 0.3	43.0 ± 0.1	48.2 ± 0.4	46.1 ± 0.6	46.1 ± 0.5
0.25	41.2 ± 0.1	38.6 ± 0.1	39.9 ± 0.1	41.7 ± 0.1	49.9 ± 0.2	45.0 ± 0.2	42.4 ± 0.1	47.4 ± 0.4	45.3 ± 0.2	46.9 ± 0.2
0.50	41.7 ± 0.1	38.8 ± 0.1	39.8 ± 0.1	42.6 ± 0.1	49.9 ± 0.2	45.1 ± 0.2	43.3 ± 0.1	49.4 ± 0.4	46.2 ± 0.1	48.2 ± 0.3
0.75	42.0 ± 0.2	39.2 ± 0.2	39.8 ± 0.2	42.9 ± 0.2	50.6 ± 0.3	46.4 ± 0.2	43.5 ± 0.1	47.7 ± 0.2	46.8 ± 0.2	48.1 ± 0.2
1.00	44.0 ± 0.2	40.3 ± 0.1	41.2 ± 0.1	43.4 ± 0.1	50.8 ± 0.2	46.6 ± 0.2	43.9 ± 0.1	50.9 ± 0.2	47.1 ± 0.1	48.5 ± 0.2
1.25	43.5 ± 0.1	40.4 ± 0.1	41.3 ± 0.2	44.3 ± 0.1	50.4 ± 0.2	47.0 ± 0.2	44.1 ± 0.1	49.9 ± 0.2	47.3 ± 0.2	49.0 ± 0.3
1.50	43.1 ± 0.2	40.4 ± 0.3	43.9 ± 0.2	44.4 ± 0.1	50.4 ± 0.4	47.8 ± 0.4	44.3 ± 0.1	50.0 ± 0.2	46.9 ± 0.2	49.1 ± 0.3

[a]Values are averages of two trials at 25 μM DNA except at 0 m cosolute which are averaged over 25 – 45 μM DNA; nonlinear regression uncertainties used for average value error propagation
[b]10 mM HEPES, 100 mmolal potassium chloride

Table S3: Observed Unfolding Enthalpies ΔH°_{obs} (kcal mol^{-1})[a] Assuming a Two-State Model for G2 Quadruplex Variants in Aqueous TMAO Solutions[b]

TMAO/mol kg^{-1}	G2	G2-TAT	G2-TCT	G2-TTT	G2-T4T4	G2-U2U2	G2-UUU	G2-U	G2-U2	G2-UUU-U2U2
0.00	41.1 ± 0.2	38.2 ± 0.2	38.8 ± 0.1	41.8 ± 0.2	49.3 ± 0.3	44.1 ± 0.3	43.0 ± 0.1	48.2 ± 0.4	46.1 ± 0.6	46.1 ± 0.5
0.25	42.3 ± 0.1	37.6 ± 0.1	38.2 ± 0.2	41.1 ± 0.1	47.7 ± 0.3	45.3 ± 0.4	43.0 ± 0.1	47.4 ± 0.1	44.9 ± 0.2	44.6 ± 0.2
0.50	41.5 ± 0.1	40.2 ± 0.5	38.3 ± 0.2	41.8 ± 0.2	49.2 ± 0.3	44.2 ± 0.2	43.0 ± 0.1	48.5 ± 0.1	46.8 ± 0.2	46.5 ± 0.2
0.75	43.6 ± 0.3	44.4 ± 0.3	40.9 ± 0.1	44.3 ± 0.1	49.2 ± 0.2	45.4 ± 0.2	43.5 ± 0.1	49.2 ± 0.1	48.5 ± 0.2	46.0 ± 0.2
1.00	47.9 ± 0.3	44.2 ± 0.2	43.2 ± 0.2	46.0 ± 0.2	53.4 ± 0.2	46.4 ± 0.2	47.5 ± 0.2	49.8 ± 0.2	49.6 ± 0.2	49.5 ± 0.3
1.25	51.9 ± 0.4	46.5 ± 0.3	45.1 ± 0.2	45.5 ± 0.1	59.9 ± 0.7	46.8 ± 0.1	51.4 ± 0.4	54.3 ± 0.4	53.4 ± 0.4	50.6 ± 0.2
1.50	54.1 ± 0.3	48.5 ± 0.5	44.8 ± 0.2	46.2 ± 0.2	54.7 ± 0.5	49.8 ± 0.3	48.3 ± 0.2	52.3 ± 0.2	51.8 ± 0.3	51.5 ± 0.3

[a]Values are averages of two trials at 25 μM DNA except at 0 m cosolute which are averaged over 25 – 45 μM DNA; nonlinear regression uncertainties used for average value error propagation
[b]10 mM HEPES, 100 mmolal potassium chloride

Table S4: Observed Unfolding Enthalpies ΔH°_{obs} (kcal mol^{-1})[a] Assuming a Two-State Model for G2 Quadruplex Variants in Aqueous Urea Solutions[b]

Urea/mol kg^{-1}	G2	G2-TAT	G2-TCT	G2-TTT	G2-T4T4	G2-U2U2	G2-UUU	G2-U	G2-U2	G2-UUU-U2U2
0.00	41.1 ± 0.2	38.2 ± 0.2	38.8 ± 0.1	41.8 ± 0.2	49.3 ± 0.3	44.1 ± 0.3	43.0 ± 0.1	48.2 ± 0.4	46.1 ± 0.6	46.1 ± 0.5
0.25	41.5 ± 0.1	38.5 ± 0.1	39.3 ± 0.1	42.2 ± 0.1	50.7 ± 0.1	45.4 ± 0.2	43.5 ± 0.2	49.5 ± 0.3	46.5 ± 0.2	45.9 ± 0.2
0.50	41.4 ± 0.1	39.0 ± 0.1	40.1 ± 0.1	43.0 ± 0.1	51.6 ± 0.2	45.8 ± 0.2	43.8 ± 0.1	49.2 ± 0.4	45.7 ± 0.2	48.0 ± 0.3
0.75	41.9 ± 0.1	39.2 ± 0.1	41.4 ± 0.1	43.3 ± 0.1	51.1 ± 0.2	47.4 ± 0.2	44.9 ± 0.1	50.6 ± 0.3	45.8 ± 0.3	49.5 ± 0.3

1.00	42.4 ± 0.1	40.5 ± 0.1	42.8 ± 0.1	44.2 ± 0.1	51.7 ± 0.2	48.7 ± 0.2	45.4 ± 0.1	52.9 ± 0.3	47.3 ± 0.2	50.7 ± 0.3
1.25	43.8 ± 0.1	41.5 ± 0.1	43.3 ± 0.1	44.8 ± 0.1	51.9 ± 0.2	49.2 ± 0.2	45.8 ± 0.1	53.3 ± 0.3	46.8 ± 0.2	52.0 ± 0.3
1.50	42.9 ± 0.2	40.7 ± 0.2	43.5 ± 0.2	45.0 ± 0.1	51.3 ± 0.3	49.0 ± 0.3	45.7 ± 0.1	53.8 ± 0.3	49.9 ± 0.3	52.1 ± 0.3

[a]Values are averages of two trials at 25 μM DNA except at 0 m cosolute which are averaged over 25 – 45 μM DNA; nonlinear regression uncertainties used for average value error propagation
[b]10 mM HEPES, 100 mmolal potassium chloride

Table S5: Transition Temperatures T_m (°C)[a] Assuming a Two-State Model for G2 Quadruplex Variants in Aqueous Glycine Betaine (GB) Solutions[b]

GB/mol kg^{-1}	G2	G2-TAT	G2-TCT	G2-TTT	G2-T4T4	G2-U2U2	G2-UUU	G2-U	G2-U2	G2-UUU-U2U2
0.00	52.36	46.14	43.63	47.74	41.62	52.91	47.66	57.44	56.05	48.05
0.25	53.63	47.81	45.11	49.33	42.02	54.20	49.25	58.42	56.68	49.46
0.50	55.08	49.55	46.70	50.71	42.91	55.37	50.55	60.06	57.86	50.67
0.75	56.36	50.92	47.93	51.97	43.39	56.61	51.50	60.27	58.41	51.64
1.00	56.40	51.37	48.49	52.38	43.78	57.69	52.18	60.42	59.64	53.17
1.25	57.18	52.10	49.62	53.27	44.18	58.69	52.97	61.26	60.36	54.12
1.50	58.11	52.99	50.65	54.10	44.77	59.43	54.00	62.15	61.14	54.76

[a]Values are averages of two trials; standard error for each temperature ± 0.14 °C determined from error propagation and an assumption of ± 0.20 °C spectrophotometer temperature resolution
[b]10 mM HEPES, 100 mmolal potassium chloride; 25 μM DNA

Table S6: Transition Temperatures T_m (°C)[a] Assuming a Two-State Model for G2 Quadruplex Variants in Aqueous Proline Solutions[b]

Proline/mol kg^{-1}	G2	G2-TAT	G2-TCT	G2-TTT	G2-T4T4	G2-U2U2	G2-UUU	G2-U	G2-U2	G2-UUU-U2U2
0.00	52.36	46.14	43.63	47.74	41.62	52.91	47.66	57.44	56.05	48.05
0.25	52.24	46.54	43.79	47.95	41.05	53.53	47.98	57.55	55.23	48.36
0.50	52.41	46.87	44.24	48.26	40.79	52.80	48.08	57.12	55.26	48.50
0.75	52.36	47.14	44.55	48.34	40.55	53.02	47.97	57.19	55.09	48.67
1.00	51.51	46.54	44.29	47.60	39.69	53.09	47.51	56.23	55.10	48.94
1.25	51.26	46.64	44.39	47.59	39.35	53.07	47.52	56.02	54.97	48.90
1.50	51.58	47.41	44.79	48.13	39.06	53.41	47.67	56.04	54.96	48.92

[a]Values are averages of two trials; standard error for each temperature ± 0.14 °C determined from error propagation and an assumption of ± 0.20 °C spectrophotometer temperature resolution
[b]10 mM HEPES, 100 mmolal potassium chloride; 25 μM DNA

Table S7: Transition Temperatures T_m (°C)[a] Assuming a Two-State Model for G2 Quadruplex Variants in Aqueous TMAO Solutions[b]

TMAO/mol kg^{-1}	G2	G2-TAT	G2-TCT	G2-TTT	G2-T4T4	G2-U2U2	G2-UUU	G2-U	G2-U2	G2-UUU-U2U2
0.00	52.36	46.14	43.63	47.74	41.62	52.91	47.66	57.44	56.05	48.05
0.25	53.36	48.27	45.32	49.43	43.16	54.90	49.40	57.56	56.73	49.68
0.50	55.48	50.19	47.17	51.03	44.57	55.88	50.75	58.86	58.06	50.99
0.75	58.07	51.71	48.19	52.09	45.72	57.37	52.10	59.92	59.34	52.26
1.00	56.46	52.37	49.30	53.03	46.34	58.74	52.65	61.43	60.64	53.48
1.25	57.12	53.60	50.14	54.47	47.65	59.68	53.13	61.71	61.24	54.39
1.50	58.30	53.40	51.67	55.73	49.16	60.71	54.84	63.31	62.92	55.70

[a]Values are averages of two trials; standard error for each temperature ± 0.14 °C determined from error propagation and an assumption of ± 0.20 °C spectrophotometer temperature resolution
[b]10 mM HEPES, 100 mmolal potassium chloride; 25 µM DNA

Table S8: Transition Temperatures T_m (°C)[a] Assuming a Two-State Model for G2 Quadruplex Variants in Aqueous Urea Solutions[b]

Urea/mol kg^{-1}	G2	G2-TAT	G2-TCT	G2-TTT	G2-T4T4	G2-U2U2	G2-UUU	G2-U	G2-U2	G2-UUU-U2U2
0.00	52.36	46.14	43.63	47.74	41.62	52.91	47.66	57.44	56.05	48.05
0.25	52.05	46.06	43.55	47.53	41.30	52.69	47.94	56.86	55.13	48.21
0.50	52.12	45.94	43.68	47.60	41.40	52.64	47.64	56.94	54.69	48.01
0.75	51.80	45.91	43.65	47.47	41.09	52.73	47.57	56.74	54.73	48.10
1.00	51.11	45.11	43.29	47.05	40.41	52.78	46.96	56.12	54.65	48.57
1.25	50.96	44.96	43.25	46.95	40.20	52.85	46.85	56.04	54.25	48.74
1.50	51.45	45.44	43.43	46.96	39.93	53.17	46.80	56.03	54.17	48.80

[a]Values are averages of two trials; standard error for each temperature ± 0.14 °C determined from error propagation and an assumption of ± 0.20 °C spectrophotometer temperature resolution
[b]10 mM HEPES, 100 mmolal potassium chloride; 25 µM DNA

Table S9: Observed Unfolding Entropies ΔS°_{obs} (cal mol^{-1} K^{-1})[a] Assuming a Two-State Model for G2 Quadruplex Variants in Aqueous Glycine Betaine (GB) Solutions[b]

GB/mol kg^{-1}	G2	G2-TAT	G2-TCT	G2-TTT	G2-T4T4	G2-U2U2	G2-UUU	G2-U	G2-U2	G2-UUU-U2U2
0.00	126 ± 1	120 ± 1	122 ± 1	130 ± 1	157 ± 1	135 ± 1	134 ± 1	146 ± 1	140 ± 2	144 ± 2
0.25	130 ± 1	122 ± 2	126 ± 1	131 ± 1	157 ± 1	138 ± 1	133 ± 1	146 ± 1	139 ± 1	144 ± 1
0.50	132 ± 1	124 ± 1	127 ± 1	134 ± 1	157 ± 1	141 ± 1	137 ± 1	145 ± 2	148 ± 1	149 ± 1
0.75	134 ± 1	127 ± 1	129 ± 1	135 ± 1	158 ± 1	142 ± 1	140 ± 1	155 ± 1	145 ± 1	152 ± 1
1.00	144 ± 1	132 ± 1	133 ± 1	140 ± 1	159 ± 1	146 ± 1	141 ± 1	156 ± 1	148 ± 1	151 ± 1
1.25	140 ± 1	133 ± 1	136 ± 1	142 ± 1	160 ± 1	148 ± 1	142 ± 1	158 ± 1	149 ± 1	154 ± 1
1.50	140 ± 1	132 ± 1	137 ± 1	143 ± 1	159 ± 1	149 ± 1	142 ± 1	159 ± 1	149 ± 1	153 ± 1

[a]Values are averages of two trials at 25 µM DNA except at 0 m cosolute which are averaged over 25 – 45 µM DNA; errors propagated from unfolding enthalpy and transition temperature uncertainties.
[b]10 mM HEPES, 100 mmolal potassium chloride

Table S10: Observed Unfolding Entropies ΔS°_{obs} (cal mol^{-1} K^{-1})[a] Assuming a Two-State Model for G2 Quadruplex Variants in Aqueous Proline Solutions[b]

Proline/mol kg^{-1}	G2	G2-TAT	G2-TCT	G2-TTT	G2-T4T4	G2-U2U2	G2-UUU	G2-U	G2-U2	G2-UUU-U2U2
0.00	126 ± 1	120 ± 1	122 ± 1	130 ± 1	157 ± 1	135 ± 1	134 ± 1	146 ± 1	140 ± 2	144 ± 2
0.25	127 ± 1	121 ± 1	126 ± 1	130 ± 1	159 ± 1	138 ± 1	132 ± 1	143 ± 1	138 ± 1	146 ± 1
0.50	128 ± 1	121 ± 1	125 ± 1	133 ± 1	159 ± 1	138 ± 1	135 ± 1	150 ± 1	141 ± 1	150 ± 1
0.75	129 ± 1	122 ± 1	125 ± 1	133 ± 1	161 ± 1	142 ± 1	135 ± 1	144 ± 1	143 ± 1	149 ± 1
1.00	136 ± 1	126 ± 1	130 ± 1	135 ± 1	162 ± 1	143 ± 1	137 ± 1	155 ± 1	143 ± 1	151 ± 1
1.25	134 ± 1	126 ± 1	130 ± 1	138 ± 1	161 ± 1	144 ± 1	138 ± 1	152 ± 1	144 ± 1	152 ± 1

1.50	133 ± 1	126 ± 1	138 ± 1	138 ± 1	161 ± 1	146 ± 1	138 ± 1	152 ± 1	143 ± 1	152 ± 1

[a]Values are averages of two trials at 25 μM DNA except at 0 m cosolute which are averaged over 25 – 45 μM DNA; errors propagated from unfolding enthalpy and transition temperature uncertainties.
[b]10 mM HEPES, 100 mmolal potassium chloride

Table S11: Observed Unfolding Entropies $\Delta S^{\circ}_{\text{obs}}$ (cal mol^{-1} K^{-1})[a] Assuming a Two-State Model for G2 Quadruplex Variants in Aqueous TMAO Solutions[b]

TMAO/mol kg^{-1}	G2	G2-TAT	G2-TCT	G2-TTT	G2-T4T4	G2-U2U2	G2-UUU	G2-U	G2-U2	G2-UUU-U2U2
0.00	126 ± 1	120 ± 1	122 ± 1	130 ± 1	157 ± 1	135 ± 1	134 ± 1	146 ± 1	140 ± 2	144 ± 2
0.25	130 ± 1	117 ± 1	120 ± 1	127 ± 1	151 ± 1	138 ± 1	133 ± 1	143 ± 1	136 ± 1	138 ± 1
0.50	126 ± 1	124 ± 2	120 ± 1	129 ± 1	155 ± 1	134 ± 1	133 ± 1	146 ± 1	141 ± 1	143 ± 1
0.75	132 ± 1	137 ± 1	127 ± 1	136 ± 1	154 ± 1	137 ± 1	134 ± 1	148 ± 1	146 ± 1	141 ± 1
1.00	145 ± 1	136 ± 1	134 ± 1	141 ± 1	167 ± 1	140 ± 1	146 ± 1	149 ± 1	149 ± 1	152 ± 1
1.25	157 ± 1	142 ± 1	140 ± 1	139 ± 1	187 ± 2	141 ± 1	158 ± 1	162 ± 1	160 ± 1	154 ± 1
1.50	163 ± 1	149 ± 2	138 ± 1	140 ± 1	170 ± 2	149 ± 1	147 ± 1	155 ± 1	154 ± 1	157 ± 1

[a]Values are averages of two trials at 25 μM DNA except at 0 m cosolute which are averaged over 25 – 45 μM DNA; errors propagated from unfolding enthalpy and transition temperature uncertainties.
[b]10 mM HEPES, 100 mmolal potassium chloride

Table S12: Observed Unfolding Entropies $\Delta S^{\circ}_{\text{obs}}$ (cal mol^{-1} K^{-1})[a] Assuming a Two-State Model for G2 Quadruplex Variants in Aqueous Urea Solutions[b]

Urea/mol kg^{-1}	G2	G2-TAT	G2-TCT	G2-TTT	G2-T4T4	G2-U2U2	G2-UUU	G2-U	G2-U2	G2-UUU-U2U2
0.00	126 ± 1	120 ± 1	122 ± 1	130 ± 1	157 ± 1	135 ± 1	134 ± 1	146 ± 1	140 ± 2	144 ± 2
0.25	128 ± 1	121 ± 1	124 ± 1	132 ± 1	161 ± 1	139 ± 1	135 ± 1	150 ± 1	142 ± 1	143 ± 1
0.50	127 ± 1	122 ± 1	127 ± 1	134 ± 1	164 ± 1	141 ± 1	137 ± 1	149 ± 1	139 ± 1	149 ± 1
0.75	129 ± 1	123 ± 1	131 ± 1	135 ± 1	163 ± 1	145 ± 1	140 ± 1	153 ± 1	140 ± 1	154 ± 1
1.00	131 ± 1	127 ± 1	135 ± 1	138 ± 1	165 ± 1	149 ± 1	142 ± 1	161 ± 1	144 ± 1	158 ± 1
1.25	135 ± 1	130 ± 1	137 ± 1	140 ± 1	166 ± 1	151 ± 1	143 ± 1	162 ± 1	143 ± 1	162 ± 1
1.50	132 ± 1	128 ± 1	137 ± 1	141 ± 1	164 ± 1	150 ± 1	143 ± 1	163 ± 1	152 ± 1	162 ± 1

[a]Values are averages of two trials at 25 μM DNA except at 0 m cosolute which are averaged over 25 – 45 μM DNA; errors propagated from unfolding enthalpy and transition temperature uncertainties.
[b]10 mM HEPES, 100 mmolal potassium chloride
